# Nrg1 Intracellular Signaling Is Neuroprotective upon Stroke

**DOI:** 10.1155/2019/3930186

**Published:** 2019-09-08

**Authors:** Carmen Navarro-González, Alba Huerga-Gómez, Pietro Fazzari

**Affiliations:** ^1^Lab of Cortical Circuits in Health and Disease, CIPF Centro de Investigación Príncipe Felipe, Valencia, Spain; ^2^Consejo Superior de Investigaciones Científicas (CSIC) Centro de Biología Molecular Severo Ochoa, Madrid, Spain

## Abstract

The schizophrenia risk gene *NRG1* controls the formation of excitatory and inhibitory synapses in cortical circuits. While the expression of different NRG1 isoforms occurs during development, adult neurons primarily express the CRD-NRG1 isoform characterized by a highly conserved intracellular domain (NRG1-ICD). We and others have demonstrated that Nrg1 intracellular signaling promotes dendrite elongation and excitatory connections during neuronal development. However, the role of Nrg1 intracellular signaling in adult neurons and pathological conditions remains largely unaddressed. Here, we investigated the role of Nrg1 intracellular signaling in neuroprotection and stroke. Our bioinformatic analysis revealed the evolutionary conservation of the NRG1-ICD and a decrease in NRG1 expression with age in the human frontal cortex. Hence, we first evaluated whether Nrg1 signaling may affect pathological hallmarks in an *in vitro* model of neuronal senescence; however, our data failed to reveal a role for Nrg1 in the activation of the stress-related pathway p38 MAPK and DNA damage. Previous studies demonstrated that the soluble EGF domain of Nrg1 alleviated brain ischemia, a pathological process involving the generation of free radicals, reactive oxygen species (ROS), and excitotoxicity. Hence, we tested the hypothesis that Nrg1 intracellular signaling could be neuroprotective in stroke. We discovered that Nrg1 expression significantly increased neuronal survival upon oxygen-glucose deprivation (OGD), an established *in vitro* model for stroke. Notably, the specific activation of Nrg1 intracellular signaling by expression of the Nrg1-ICD protected neurons from OGD. Additionally, time-lapse experiments confirmed that Nrg1 intracellular signaling increased the survival of neurons exposed to OGD. Finally, we investigated the relevance of Nrg1 intracellular signaling in stroke *in vivo*. Using viral vectors, we expressed the Nrg1-ICD in cortical neurons and subsequently challenged them by a focal hemorrhagic stroke; our data indicated that Nrg1 intracellular signaling improved neuronal survival in the infarcted area. Altogether, these data highlight Nrg1 intracellular signaling as neuroprotective upon ischemic lesion both *in vitro* and *in vivo*. Given the complexity of the neurotoxic effects of stroke and the involvement of various mechanisms, such as the generation of ROS, excitotoxicity, and inflammation, further studies are required to determine the molecular bases of the neuroprotective effect of Nrg1 intracellular signaling. In conclusion, our research highlights the stimulation of Nrg1 intracellular signaling as a promising target for cortical stroke treatment.

## 1. Introduction

The major synaptogenic protein Neuregulin 1 (Nrg1) controls the formation of excitatory and inhibitory synapses in the cortex [1–3]. The *Nrg1* gene encodes more than 20 isoforms grouped into six types of proteins. While the Nrg1 extracellular domain displays high variability, all isoforms contain the epidermal growth factor- (EGF-) like domain located in the extracellular domain that is necessary and sufficient for activation of the ErbB4 receptor. In addition, many Nrg1 isoforms possess common transmembrane and intracellular domains that elicit Nrg1 intracellular (or noncanonical) signaling [1]. Similarly to Notch signaling, the transmembrane domain of Nrg1 is first cleaved by alpha- or beta-secretases and subsequently by gamma-secretase [4, 5]. Neuronal activity or binding to the ErbB4 receptor triggers this regulated processing [2, 6–8], releasing the resulting intracellular domain of Nrg1 (Nrg1-ICD) for translocation to the nucleus.

Different studies have demonstrated that Nrg1/ErbB4 canonical signaling controls both the formation of cortical inhibitory circuits and the synchronization of neuronal activity [1, 3, 9–12]. Many of these studies used gain- and loss-of-function approaches to investigate the role of the ErbB4 receptor and its canonical signaling in inhibitory interneurons. Given the strong association of Nrg1 with schizophrenia [1], most studies have focused on the neurodevelopmental role of Nrg1 signaling in the formation of cortical circuits. In this context, we and others have established that Nrg1 intracellular signaling promotes neurite development and the establishment of excitatory synapses [2, 5, 7]. During the development of the peripheral nervous system, Nrg1 stimulates myelination and promotes survival of sensory neurons [1, 6, 13]. In addition, studies have implicated Nrg1 in pathologies such as neuroinflammation [14–17], neurodegenerative disorders [18–20], and stroke [21–24]. However, the role of noncanonical Nrg1 intracellular signaling in pathological conditions or mature neurons remains largely unaddressed. Of note, CRD-Nrg1 (also known as type III Nrg1), a transmembrane isoform endowed with intracellular signaling, represents the most abundant isoform of Nrg1 in adult neurons.

Herein, we investigated the role of Nrg1-ICD signaling in mature neurons and tested a working hypothesis that this pathway may exert a neuroprotective role in mature cortical neurons. As our bioinformatic query revealed a decrease in NRG1 expression with age, we first hypothesized a link between Nrg1 intracellular signaling and aging. In our *in vitro* experiments, Nrg1 intracellular signaling failed to affect the tested hallmarks of neuronal senescence, namely, DNA damage and p38 mitogen-activated protein kinase (MAPK) activation, a pathway activated by age-related stressors such as inflammation and oxidative stress.

As previous studies indicated an increase in Nrg1 expression in response to hypoxia and that ErbB4 activation reduced ischemic infarct, we hypothesized that Nrg1 intracellular signaling might be neuroprotective in hypoxia and stroke.

Our data revealed that OGD conditions triggered the activation of Nrg1 intracellular signaling in cortical neurons *in vitro*, while the activation of Nrg1 intracellular signaling reduced neuronal death following OGD. Notably, we discovered that Nrg1 also improved neuronal survival in an experimental model of cortical hemorrhagic stroke *in vivo*.

## 2. Materials and Methods

### 2.1. Bioinformatic Analyses of the Phylogenetic Conservation of the Nrg1 Transmembrane Region and NRG1 Expression

The sequences of *NRG1* were downloaded from the Ensembl website (https://www.ensembl.org) and aligned using ClustalW (McGettigan PA 2007). We aligned the following sequences: panda (Ailuropoda melanoleuca; ENSAMEP00000014935), dog (Canis familiaris; ENSCAFP00000009544), cat (Felis catus; ENSFCAP00000018373), ferret (Mustela putorius; ENSMPUP00000010121), chimpanzee (Pan troglodytes; ENSPTRP00000034489), human (homo sapiens; ENSP00000287842), gorilla (Gorilla; ENSGGOP00000023096), Macacus (Macaca mulatta; ENSMMUP00000000296), marmoset (Callithrix jacchus; ENSCJAP00000047313), cavia (Cavia porcellus; ENSCPOP00000007007), mouse (Mus musculus; ENSMUSP00000073546), and sheep (Ovis aries; ENSOARP00000000211). The meta-analysis of NRG1 expression in human aging was described previously [25]. The meta-analysis database is publicly available under Creative Commons CC BY-NC-ND 4.0 license [25].

### 2.2. NRG1 Gene and Protein Naming

We followed the naming of Neuregulin 1 used in NCBI and Ensembl websites for the use of capital letters and the NCBI Style Guide for the use of italic and nonitalic letters. Therefore, we used the following: *NRG1*, for the human gene; NRG1, for the human protein; *Nrg1*, for the mouse gene; and Nrg1, for the mouse protein.

### 2.3. Nrg1 Constructs and Adeno-Associated Viral Vectors

The original constructs for the expression of GFP-tagged Nrg1-ICD and CRD-Nrg1 (Nrg1-FL), aka type III Nrg1, from *Mus musculus*, were previously generated and fully described [5]. The constructs were subcloned into the pAAV-hSyn-hChR2(H134R)-mCherry vector kindly deposited by Karl Diesseroth into the Addgene repository (https://www.addgene.org/26976). Briefly, the original open reading frame was excised and the Nrg1 sequence was inserted after the human Synapsin promoter. Correct cloning was verified via sequencing. The vector for GFP expression under the human Synapsin promoter was kindly deposited into the Addgene repository by Bryan Roth (https://www.addgene.org/50465). The adeno-associated viral particles (AAVs), serotype 1, were produced and purchased by the Viral Vector Production Unit (UPV) of the Universitat Autonoma de Barcelona, Spain, according to standard protocols. The physical titer of the viral particle was also evaluated by immunofluorescence in neurons to assess the biological activity and obtain the same level of infection for the three viruses. The correct expression of the constructs was also verified by Western blot (not shown).

### 2.4. Neuronal Cultures

Primary cultures of cortical neurons were prepared from embryonic day 17-18 (E17-18) C57Bl6J mice, as previously described [3, 5]. Briefly, embryonic brains were dissected and placed into ice-cold Hank's solution with 7 mM HEPES and 0.45% glucose. The tissue was trypsinized at 37°C for 15 min and then treated with DNase (72 *μ*g mL^−1^; Sigma-Aldrich) for 1 min at 37°C. Cortices were washed Hank's solution, dissociated by mechanical disaggregation in 5 mL of plating medium (Minimum Essential Medium (MEM) supplemented with 10% horse serum and 20% glucose), and counted in a Neubauer chamber. Cells were plated into precoated dishes with poly D-lysine (Sigma-Aldrich) (150,000 cells per well in 12-well plates) and placed into a humidified incubator containing 95% air and 5% CO_2_. The plating medium was replaced with equilibrated neurobasal media supplemented with B27 and GlutaMAX (Gibco; Life Technologies Co.). Infection with viral vectors was performed on day 3 (D3). On D10, the culture medium was replaced with medium without GlutaMAX. For the paradigm of *in vitro* aging, the culture medium was substituted at D14 with a medium with B27 minus antioxidants (Thermo Fisher, 10889038) and cells were analyzed at D21.

Oxygen-glucose deprivation (OGD) was performed as previously described [26]. Briefly, the OGD medium (1 mM CaCl_2_, 5 mM KCl, 137 mM NaCl, 0.4 mM KH_2_ PO_4_, 0.3 mM Na_2_HPO_4_, 0.5 mM MgCl_2_, 0.4 mM MgSO_4_, 25 mM HEPES, 4 mM NaHCO_3_) was equilibrated at 37°C and saturated with bubbling nitrogen. The culture medium was substituted with the saturated OGD medium in a custom-made hypoxia chamber at conditions with 0% oxygen. After the hypoxic stress, the OGD medium was changed again for the original conditioned culture medium.

### 2.5. Immunofluorescence

Immunocytochemistry was performed according to a standard protocol as previously described [5]. Briefly, neurons were fixed with 4% paraformaldehyde for 10 min, permeabilized for 10 min with PBS with 0.1% Triton, and then blocked with 2% PBS-BSA. Primary and secondary antibodies were diluted in 2% PBS-BSA. After staining, the coverslips were mounted in Mowiol for imaging. For fixed material, pictures were taken with an Axioskop 2 (Zeiss) microscope and a CoolSNAP FX camera (Roper Scientific): objectives 10X/0.3 Plan-Neofluar, 25X/0.8 Korr Plan-Neofluar Ph2 DICIII oil. Time-lapse imaging used a microscope Axiovert 200 (Zeiss), a camera ORCA-Flash4.0 LT sCMOS (C11440-42U) (Hamamatsu), and an objective 10X/0.3 Plan-Neofluar. Images were processed and analyzed with ImageJ and GIMP and mounted with Inkscape software. GraphPad was used for statistical analysis and generation of graphs.

The images of the nuclear localization of Nrg1-ICD upon OGD were acquired with a Leica TCS SP2 AOBS (Leica Microsystems Heidelberg GmbH, Mannheim, Germany) inverted laser scanning confocal microscope using the oil objective 63X Plan-Apochromat-Lambda Blue 1.4 N.A. The excitation wavelengths for fluorochromes were 488 nm (argon laser) for GFP and 405 nm (blue diode) for DAPI. Two-dimensional pseudocolor images were gathered with a size of 1024 × 1024 pixels and Airy 1 pinhole diameter. All confocal images were acquired using the same settings. ImageJ was used to analyze the confocal images. First, a Z-stack was generated and an image showing a nuclear plane was selected for each stack. GFP fluorescence intensity was measured in the soma and in the nucleus of each neuron using DAPI staining as a mask for delimiting the nucleus. A total of 27 neurons (9 neurons per well) were analyzed in three independent wells per each experimental condition.

Antibodies used were phopsho-p38 MAPK (1/200; sc-166182; Santa Cruz), phospho-histone-h2a-x-ser139-antibody, phospho-H2AX (1/200; 2577, Cell Signaling Technology (CST)), GFP (1/500; GFP-1020, Aves Labs), and MAP2 (1/500; MA5-12826, Thermo Fisher). All secondary antibodies are Alexa conjugated from Life Technologies.

### 2.6. RNA Extraction and qPCR

The neurons were cultured in normoxic conditions and analyzed at D14. Neuronal cultures were homogenized with TRIzol Reagent (Ambion/RNA Life Technologies Co.), and RNA was extracted with Direct-zolTM RNA minipreps (ZIMO research ref. R2052) following the manufacturer's instructions. RNA was quantified by absorbance at 260 nm using a NanoDrop ND-100 (Thermo Scientific; Thermo Fisher Scientific Inc.). Retrotranscription to first-strand cDNA was performed using a RevertAid H Minus First Strand cDNA Synthesis Kit (Thermo Scientific; Thermo Fisher Scientific Inc.). Briefly, 5 ng of synthesized cDNA was used to perform the qPCR using GoTaq® qPCR Master Mix (Promega Co., Madison, WI, USA) in ABI PRISM 7900HT SDS (Applied Biosystems; Life Technologies Co.). All values were normalized with the housekeeping gene Gapdh. The primer pairs used in this study were designed and validated by PrimerBank (https://pga.mgh.harvard.edu/primerbank/) [27].

Primer sequences:

Casp2 (GGAGCAGGATTTTGGCAGTGT, GCCTGGGGTCCTCTCTTTG)

Casp3 (ATGGAGAACAACAAAACCTCAGT, TTGCTCCCATGTATGGTCTTTAC)

Casp7 (CCCACTTATCTGTACCGCATG, GGTTTTGGAAGCACTTGAAGAG)

DAPK1 (ATGACTGTGTTCAGGCAGGAA, CCGGTACTTTTCTCACGACATTT)

Apaf1 (AGTGGCAAGGACACAGATGG, GGCTTCCGCAGCTAACACA)

MAPK1 (GGTTGTTCCCAAATGCTGACT, CAACTTCAATCCTCTTGTGAGGG)

BAD (AAGTCCGATCCCGGAATCC, GCTCACTCGGCTCAAACTCT)

BAX (TGAAGACAGGGGCCTTTTTG, AATTCGCCGGAGACACTCG)

Bcl-2 (GTCGCTACCGTCGTGACTTC, CAGACATGCACCTACCCAGC)

BCL2I1 (GACAAGGAGATGCAGGTATTGG, TCCCGTAGAGATCCACAAAAGT)

Akt1 (ATGAACGACGTAGCCATTGTG, TTGTAGCCAATAAAGGTGCCAT)

MAPK14 (TGACCCTTATGACCAGTCCTTT, GTCAGGCTCTTCCACTCATCTAT)

Rbfox3 (ATCGTAGAGGGACGGAAAATTGA, GTTCCCAGGCTTCTTATTGGTC)

Gapdh (CTCCCACTCTTCCACCTTCG, CATACCAGGAAATGAGCTTGACAA)

### 2.7. Experimental *In Vivo* Model of Stroke

The experiments were performed in 3-month-old C57Bl6J female mice provided by Charles River at the Centro de Biología Molecular “Severo Ochoa” (CSIC), Madrid, Spain. Experiments were supervised by the bioethics committee of the institute and performed in compliance with bioethical regulations of the European Commission. Animals were group housed with food and water ad libitum.

The stereotactic injection of viral vectors for the expression of GFP and Nrg1-ICD was performed 30 days before experimental stroke. The operation was performed according to standard procedure as previously described [28]. Briefly, mice were anesthetized with isoflurane and placed in a stereotaxic frame. Coordinates (mm) relative to bregma were as follows: anteroposterior, 1.3; mediolateral, 1.0; and dorsoventral axes, 0.8. 1 *μ*L of the virus was injected at a flow rate of 0.2 *μ*L per minute with a Hamilton syringe. At the end of the injection, the needle was left in place for 5 minutes to allow the diffusion of the virus and then gently withdrawn. The injection of collagenase was performed with a similar procedure: 18 mU of collagenase (from Clostridium histolyticum, type VII-S, catalog number C2399, Sigma-Aldrich) in a volume of 1 *μ*L was injected at stereotaxic coordinates relative to bregma as follows: anteroposterior, 1.7; mediolateral, 1.0; and dorsoventral axes, 0.8. 1 *μ*L of collagenase was injected at a flow rate of 0.2 *μ*L per minute with a Hamilton syringe. At the end of the injection, the needle was left in place for 5 minutes to allow the diffusion of the collagenase and then slowly withdrawn. Mice were allowed to recover and sacrificed 24 hours after the injection of the collagenase.

### 2.8. Histology

Mouse brains were fixed and processed as previously described [29]. Briefly, mice were perfused through the circulatory system with 4% paraformaldehyde, postfixed for 2 hours, cryoprotected in 30% sucrose, and then cut with a cryostat at 40 *μ*m. Primary and secondary antibodies were diluted in PBS with 0.25% Triton and 4% BSA and incubated overnight in floating sections. The antibody for GFP staining (GFP-1020, Aves Lab) was diluted 1/500. The Alexa488-conjugated anti-chicken secondary antibody was purchased from Life Technologies. Pictures were taken with a Leica DM6000B microscope equipped with a Leica DFC35FX camera and an HCX PL FLUOTAR 10X 0.3 dry objective.

## 3. Results

### 3.1. NRG1 Phylogenetic Conservation and Expression during Human Aging

The *NRG1* gene generates six types of protein and more than 20 isoforms that are differentially expressed during development and regulated by neuronal activity [1, 30]. These isoforms mostly diverge in the extracellular region, while the transmembrane and the ICD are common to the majority of isoforms. Therefore, we investigated the phylogenetic conservation of NRG1 with a specific focus on the transmembrane domain (TM) and the initial section of the ICD. These regions contain sequences crucial to the intracellular signaling of NRG1 as they control NRG1 processing and ICD nuclear localization.

**Figure 1:**
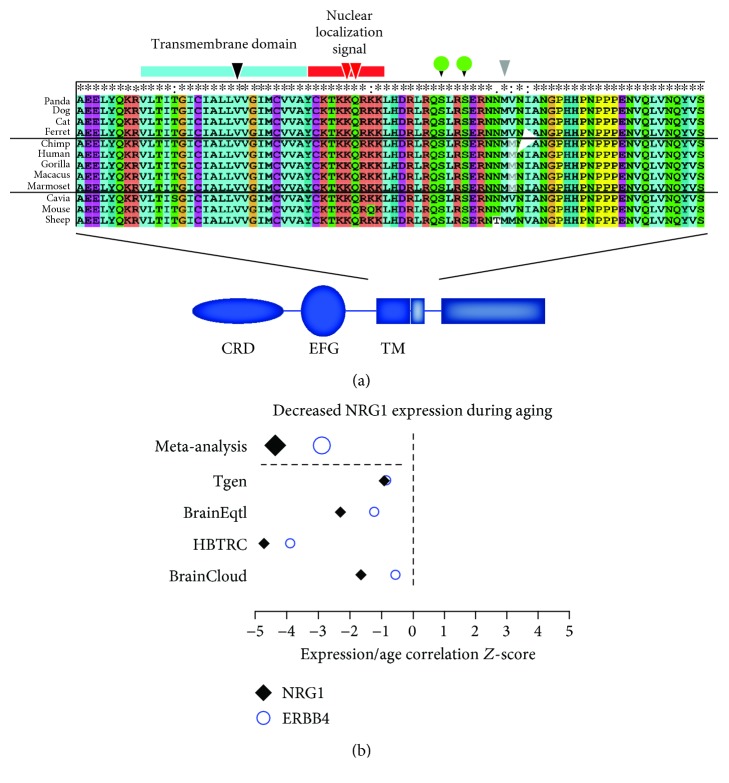
NRG1 phylogenetic conservation and expression in aging. (a) Sequence alignment of the transmembrane and intracellular regions of NRG1 that are crucial for intracellular signaling, namely, the transmembrane domain (in light-blue), the nuclear localization signal (in red), and the phosphorylation sites (green circles). The black arrowhead indicates the location of the Val-to-Leu (rs7494201) schizophrenia-linked mutation that affects gamma-secretase processing and Nrg1 intracellular signaling. The red arrowheads indicate the amino acids in the nuclear localization signal required for the nuclear targeting of Nrg1-ICD. The grey arrowhead indicates the site of the Met-to-Thr mutation, rs10503929, while the white arrowhead indicates a primate-specific polymorphism. CRD: cysteine-rich domain; EGF: epithelial growth factor domain; TM: transmembrane domain. (b) Forest plot showing the correlation *Z*-score between age and mRNA expression for NRG1 and ERBB4 in four independent datasets (Tgen, *n* = 179; BrainEqtl, *n* = 256; HBTRC, *n* = 153; BrainCloud, *n* = 128). Meta-analysis conducted at the gene level employing Stouffer's weighted *Z*-score.

We compared NRG1 sequences in different mammals, including mouse, human, and four nonhuman primates. Notably, we discovered the full conservation of the amino acids of the TM domain and the phosphorylation sites that control the processing of NRG1 in the species tested [4, 7, 31, 32], as well as the nuclear localization signal. Interestingly, a methionine whose mutation has been linked to schizophrenia (Met-to-Thr, rs10503929) [33, 34] displayed full conservation and precedes another methionine conserved in all primates, while not being found in the other mammals tested (Figure 1(a)). Overall, the phylogenetic conservation of amino acids crucial for Nrg1 intracellular signaling further supports the relevance of the Nrg1-ICD signaling pathway.

Recent studies suggested the involvement of NRG1 in age-related neurodegenerative disorders [18–20] and the decrease of NRG1 expression during aging [30]. As the analysis of NRG1 expression in humans was performed on a small dataset, we decided to perform the analysis of NRG1 expression in larger independent datasets. Specifically, we queried the expression of NRG1 and its putative receptor ERBB4 in a database that reports the meta-analysis of transcriptome-wide microarray datasets from four independent human cohorts, namely, Tgen, BrainEqtl, HBTRC, and BrainCloud [25]. In total, these databases provided *n* = 716individuals > 25 years of age. In all four cohorts, there existed a negative correlation between age and NRG1/ERBB4 expression, indicating a decrease in both NRG1 and ERBB4 expression with increasing age (Figure 1(b)). The decrease in expression was particularly evident for NRG1 as confirmed by the meta-analysis of the four databases (NRG1, *p* = 1.69*E* − 05; false discovery rate *p* = 0.000403; ERBB4, *p* = 0.00361; false discovery rate *p* = 0.0303).

We reasoned that the conservation of NRG1-ICD signaling, the decreased expression of NRG1/ERBB4 during aging, and the involvement of NRG1 in neurodegeneration might indicate a role of NRG1 intracellular signaling in neuroprotection in the aging brain.

To address this working hypothesis, we first generated viral vectors to express CRD-Nrg1 full-length (Nrg1-FL) and Nrg1-ICD.

### 3.2. Generation and Validation of Viral Vectors Expressing Nrg1

**Figure 2:**
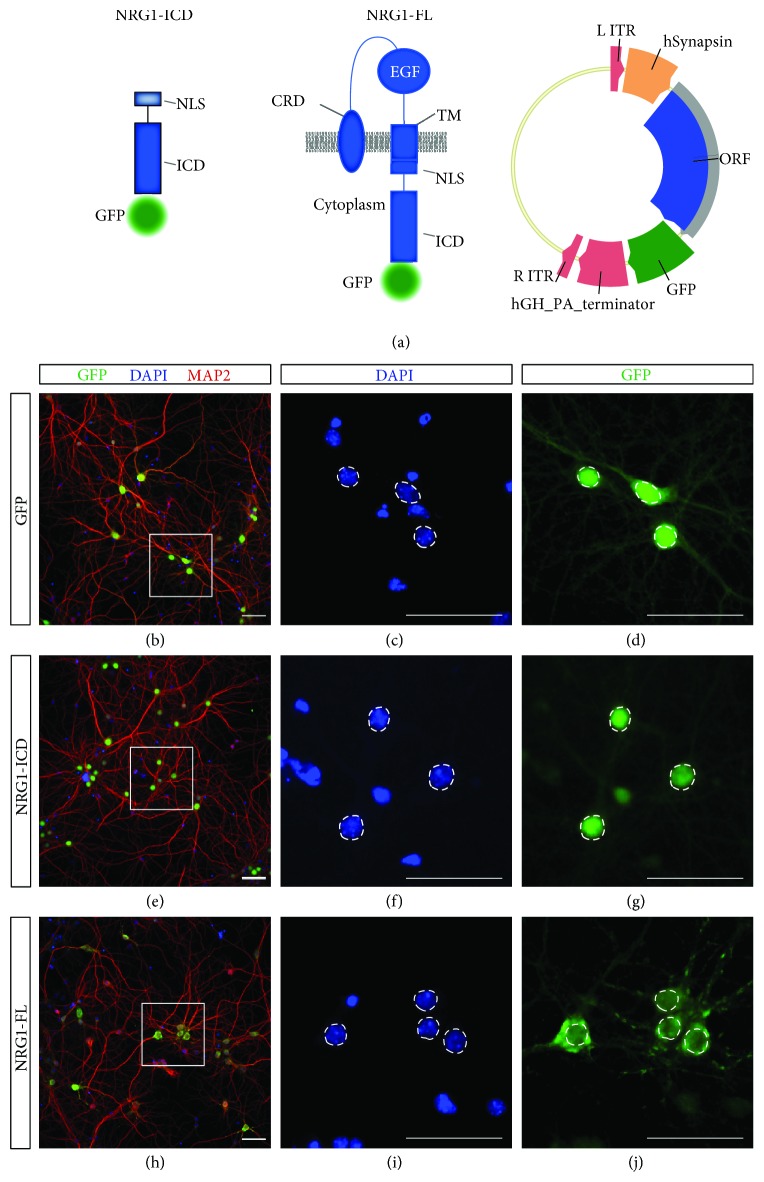
Generation and validation of viral vectors for Nrg1 expression. (a) Schematic representation of the constructs for the expression of Nrg1-ICD (including the nuclear localization signal (NLS)) and Nrg1-FL both fused to GFP. The schema of the vector for the production of AAV particles to express Nrg1 under human Synapsin promoter is depicted on the right. CRD: cysteine-rich domain; EGF: epithelial growth factor domain; TM: transmembrane domain; ITR: inverted terminal repeat; ORF: open reading frame; hGH PA: human growth hormone polyadenylation signal. (b–j) Immunofluorescent labeling of primary cortical neurons infected to express GFP, Nrg1-ICD, or Nrg1-FL and fixed at D14. The neurons were labeled with DAPI and microtubule-associated protein 2 (MAP2) to visualize neuronal dendrites. The boxed area in (b, e, h) depicts the area magnified in (d, g, j). The dotted lines delimit the nuclei to highlight the specific localization of Nrg1 constructs. Scale bar, 50 *μ*m.

We and others have previously established that the expression of the Nrg1-ICD mimics the end product of CRD-Nrg1 full-length processing by gamma-secretase and consistently activates Nrg1 intracellular signaling [5, 6]. To improve upon this approach, we generated AAVs to express Nrg1-ICD and CRD-Nrg1 full-length (hereafter, Nrg1-FL) under the human Synapsin promoter (Figure 2(a)). We discovered that these vectors allowed the efficient and neuronal-specific expression of Nrg1. We fused the C-terminal portion of Nrg1 to GFP to enable visualization, and as expected, the Nrg1-ICD construct localized mainly in the nucleus while the Nrg1-FL localizes mostly to the cytosol and membrane. The neurons expressing the Nrg1-ICD and Nrg1-FL failed to exhibit overt signs of anomalies when compared to GFP-expressing control neurons (Figure 2(b)).

### 3.3. Nrg1 Intracellular Signaling Does Not Affect p38 MAPK Activation and DNA Damage *In Vitro*

Next, we began to evaluate the working hypothesis that Nrg1 intracellular signaling may be neuroprotective during neuronal aging *in vitro*. In long-term culture, neurons accumulate various alterations related to senescence, including oxidative stress, DNA damage, and changes in lipid composition [35–37].

**Figure 3:**
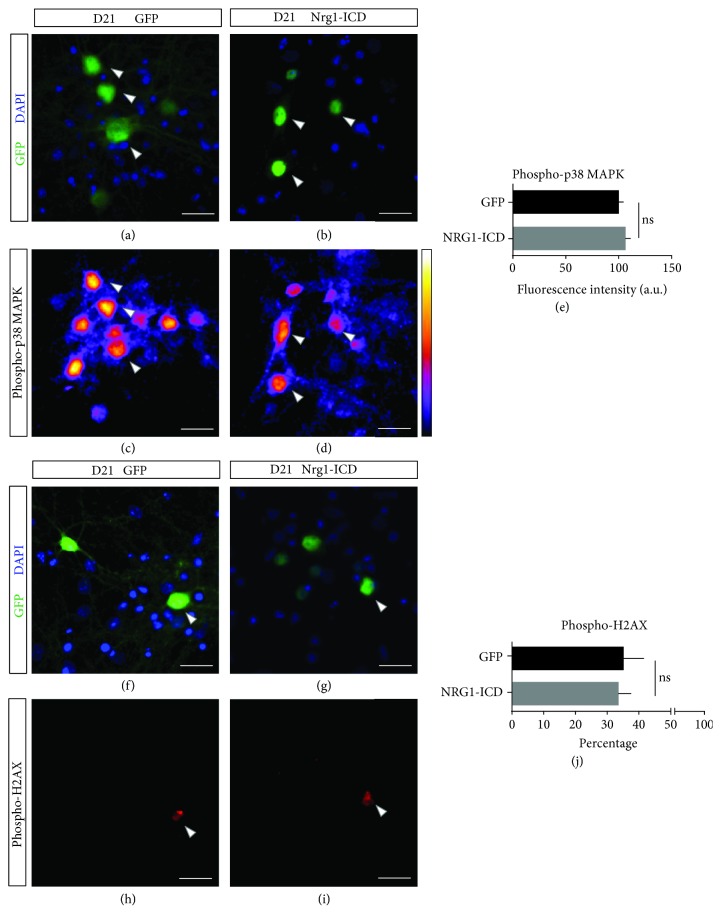
Nrg1 intracellular signaling does not affect the activation of p38 MAPK and DNA damage *in vitro.* (a–d) Representative images of neurons subjected to *in vitro* aging expressing either GFP as control or Nrg1-ICD labeled by GFP (a, b) and phospho-p38 MAPK (c, d). The color bar on the right illustrates the lookup table (LUT) for signal intensity. Scale bar, 20 *μ*m. (e) Graph illustrates the quantification of phospho-p38 MAPK labeling expressed in arbitrary units (a.u.). *n* > 9 fields from two neuronal cultures. *p* = 0.325, *t*-test. Average ± sem. (f–i) Representative images of aged primary neurons treated as in (a–d). Labeling shows GFP (f, g) and phospho-H2AX (h, i). Scale bar, 20 *μ*m. (j) The graph shows the percentage of GFP or Nrg1-ICD-expressing neurons positive for phospho-H2AX. *n* = 10 fields from two neuronal cultures. *p* = 0.833, *t*-test. Average ± sem.

Here, we focused on two hallmarks of neuronal senescence: the activation of the stress sensor p38 MAPK and DNA damage (Figure 3 and Supplemental [Supplementary-material supplementary-material-1]). Different cellular stressors, such as inflammatory cues and oxidative stress, activate p38 MAPK [38]. As a readout for p38 activation, we measured the phosphorylation of p38 MAPK, and to measure DNA damage, we quantified the phosphorylation of H2AX, a canonical marker of DNA damage [39].

We cultured cortical neurons for two weeks under standard conditions to allow cortical neuron maturation [5]. At D14 in our experimental conditions, we observed a low level of phosphorylated p38 MAPK with only a few neurons displaying signs of DNA damage (Supplemental [Supplementary-material supplementary-material-1]). After D14, we cultured cortical neurons in a specific medium lacking antioxidants to accelerate senescence and then analyzed neurons at D21. These conditions prompted a robust increase in both p38 MAPK activation and DNA damage; however, we failed to encounter any significant differences between Nrg1-ICD- and GFP-expressing neurons regarding the activation of p38 MAPK or the presence of DNA damage at D21 (Figure 3). While we acknowledge the caveats and limitations inherent in this experimental paradigm, our findings do not seem to indicate a significant role of Nrg1 in the regulation of the hallmarks that we tested.

### 3.4. OGD Stimulates Nrg1 Intracellular Signaling *In Vitro*

Previous studies suggested that Nrg1 forward signaling via ErbB4 may aid recovery from stroke [21, 22, 40], a pathology associated with aging in humans [41]. As the involvement of Nrg1 intracellular signaling in stroke remains unaddressed, we assessed whether Nrg1 intracellular signaling could be neuroprotective in stroke.

**Figure 4:**
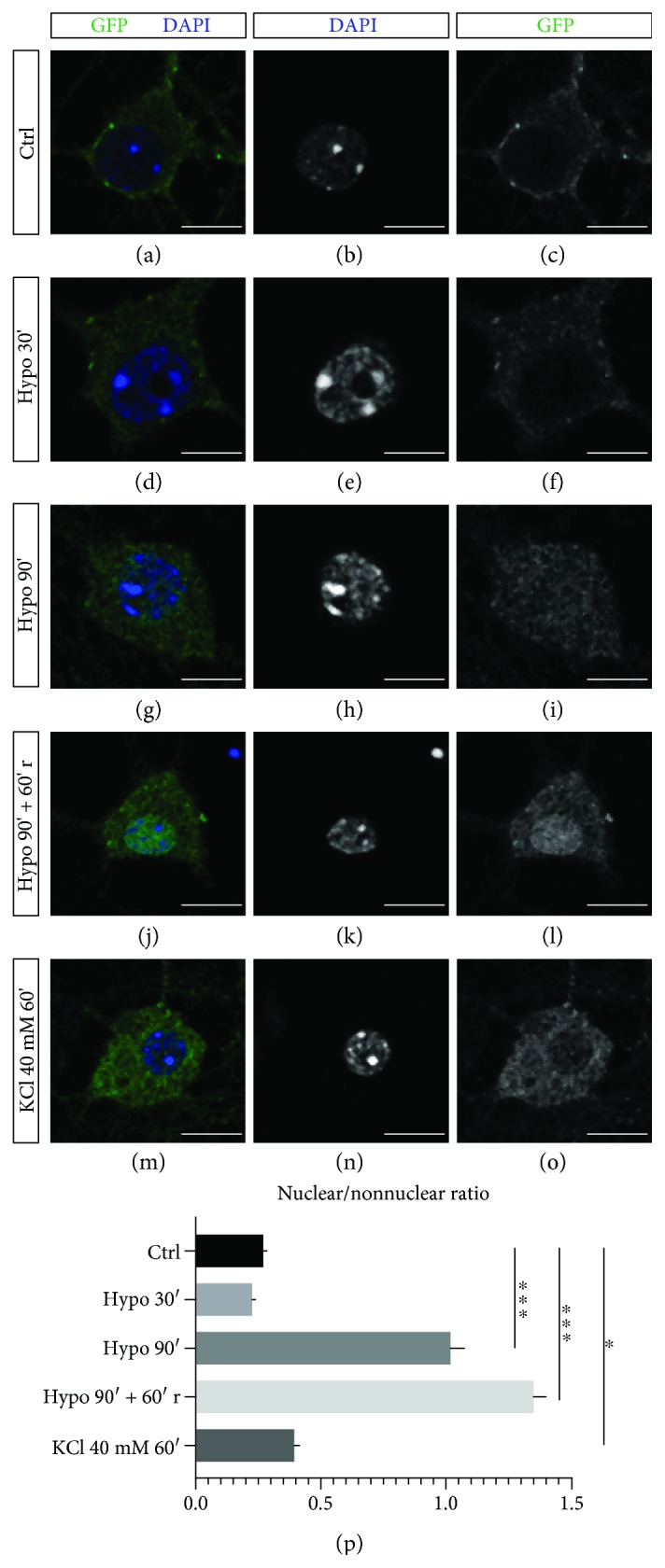
OGD stimulates Nrg1 intracellular signaling *in vitro*. (a) Representative images of neurons expressing Nrg1 tagged with GFP in its C-terminal domain. (a–c) represent the normoxic control, and (d–o) depict images of neurons after hypoxia. OGD was performed at D14, and the neurons were fixed immediately after 30 or 90 minutes of OGD or after 60 minutes of recovery after OGD. Treatment with 40 mM of KCl for 1 hour was used as a positive control. Scale bar, 10 *μ*m. (p) The graph illustrates the ratio of the quantification of GFP fluorescence intensity in the cell nucleus (Nrg1-ICD) to that in the soma (Nrg1-FL). *n* = 3. Average ± sem. ^∗^*p* < 0.05, ^∗∗∗^*p* < 0.001, one-way ANOVA, and Tukey's multiple comparisons test. Average ± sem.

We first asked whether OGD conditions altered Nrg1 processing and the activation of Nrg1 intracellular signaling. Neuronal depolarization or binding to the receptor ErbB4 triggers Nrg1 intracellular signaling; the transmembrane domain of Nrg1 becomes cleaved, and this processing releases the ICD that then translocates to the nucleus [2, 5–7]. With this in mind, we infected cortical neurons to express Nrg1-FL tagged with GFP at the C-terminus to follow the nuclear localization of Nrg1-ICD. Neuronal depolarization induced by KCl treatment increased the nuclear localization of Nrg1-ICD similarly to that previously reported for spiral ganglion neurons [6]. Also, we challenged D14 neurons with OGD, an established in vitro model for neuronal stroke, revealing a marked increase in Nrg1 nuclear localization upon 90 minutes of OGD, and after 60 minutes of recovery from OGD (Figure 4).

These data indicate that OGD conditions stimulate Nrg1 intracellular signaling in cortical neurons, and this finding prompted us to evaluate the functional relevance of Nrg1 intracellular signaling during OGD.

### 3.5. Nrg1 Intracellular Signaling Hampers Neuronal Loss in an *In Vitro* Model of Stroke

**Figure 5:**
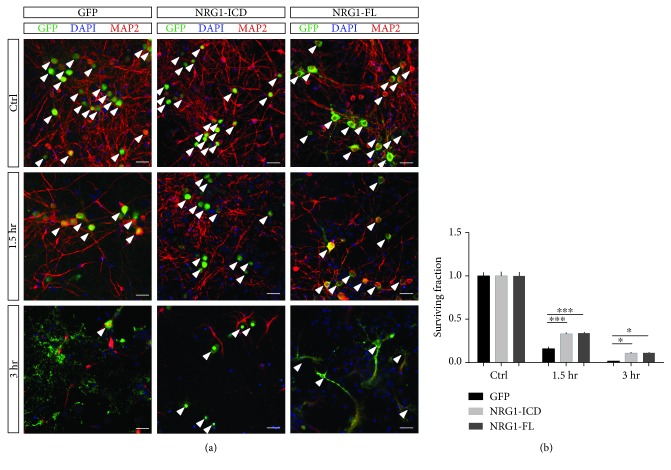
Nrg1 intracellular signaling inhibits neuronal loss in an *in vitro* model of stroke. (a) Representative images of neurons labeled for GFP and MAP2. The neurons were infected to express either GFP as control or Nrg1-ICD or Nrg1-FL (both Nrg1 constructs are tagged with GFP). OGD was performed at D14, and the neurons were fixed 24 hours after OGD. Arrowheads indicate the surviving neurons. Scale bar, 50 *μ*m. (b) The graph illustrates the quantification of neuronal survival upon OGD compared to control conditions. *n* = 25 fields out of three cultures. ^∗∗∗^*p* < 0.001, ^∗^*p* < 0.05, two-way ANOVA, and Dunnett's multiple comparisons test. Average ± sem.

We cultured primary cortical neurons infected with AAVs to express either Nrg1-ICD, Nrg1-FL, or GFP as a control (Figure 5). We challenged D14 neurons with OGD and quantified neuronal survival 24 hours after insult.

Histologically, stroke is characterized by a “core” and by a peri-infarct zone termed the “penumbra” that display differing degrees of neuronal loss [41]. To evaluate the role of Nrg1 intracellular signaling, we challenged the neurons with OGD for 1.5 and 3 hours. Given the GFP tagging of Nrg1-ICD and Nrg1-FL, we directly quantified the fractions of surviving neurons expressing Nrg1 as compared to GFP-expressing controls. We discovered that both the expression of Nrg1-FL and the specific activation of Nrg1 intracellular signaling improved the survival of neurons exposed to OGD when compared to controls (Figure 5). Notably, the activation of Nrg1 intracellular signaling by the expression of Nrg1-ICD hampered neuronal loss in this experimental paradigm to a similar extent. Furthermore, while we observed damage to the integrity of neuritic arborization and alterations to the morphology of surviving neurons, the Nrg1-ICD and Nrg1-FL partly alleviated neurodegeneration induced by 3 hours of OGD when compared to control.

Taken together, the data gathered from this *in vitro* model suggest a neuroprotective role of Nrg1 intracellular signaling upon stroke.

### 3.6. Nrg1 Intracellular Signaling Delays the Onset of Neurodegeneration

**Figure 6:**
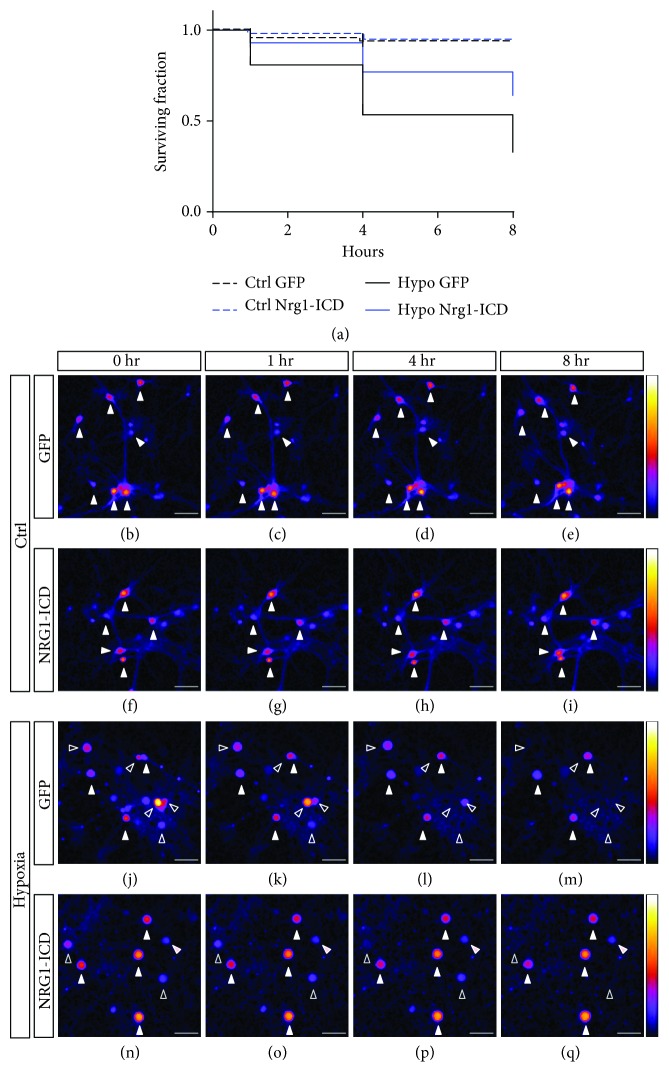
Nrg1 intracellular signaling delays the onset of neurodegeneration. (a) Kaplan-Meier plot displays the surviving fractions of neurons expressing Nrg1-ICD in control and OGD conditions when compared to GFP-expressing neurons. After OGD, neurons were imaged for 8 hours. *n* > 52 per group. Gehan-Breslow-Wilcoxon test for comparison between GFP and Nrg1-ICD upon OGD, *p* < 0.001. (b–q) Representative images of the Nrg1-ICD and GFP plotted in (a). (b–i) display normoxic controls, and (j–q) display neurons imaged after OGD at different time points. The LUT bar is shown on the left-hand side. Full arrowheads indicate neurons that survived throughout the time-lapse. Empty arrowheads depict neurons that died within 8 hours after OGD. The right bar shows the LUT. Scale bar, 50 *μ*m.

To gain further insight into the neuroprotective role of Nrg1-ICD, we performed time-lapse experiments on neurons subjected to OGD. Briefly, we coinfected primary neuronal cultures to express Nrg1-ICD with GFP to allow the visualization of live neurons or only GFP as control. At D14, we subjected neurons to OGD for 2 hours and then filmed the neurons for 8 hours after the OGD. Under these experimental conditions, the activation of Nrg1 intracellular signaling significantly delayed the onset of neuronal death starting from early time points. The difference in neuronal survival between control and Nrg1-expressing neurons further increased at 4 and 8 hours after OGD (Figure 6).

These data also support the neuroprotective role of Nrg1-ICD *in vitro* upon OGD, suggesting that Nrg1 intracellular signaling hinders neurodegeneration.

### 3.7. Nrg1 Intracellular Signaling Controls the Expression of Apoptotic Genes

**Figure 7:**
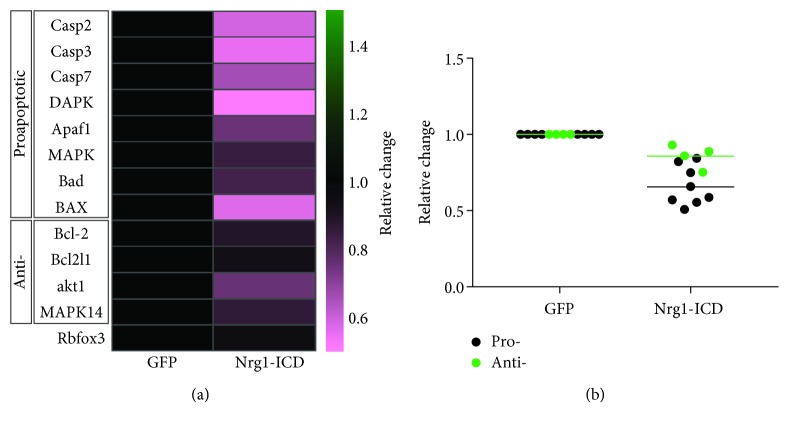
Nrg1 intracellular signaling controls the expression of apoptosis-related genes. (a) The heatmap displays the expression of candidate pro- and antiapoptotic genes evaluated in Nrg1-ICD-expressing neurons when compared to GFP controls. The neuronal marker Rbfox3 is highlighted in blue. The right bar shows the LUT for the heatmap expressed in fold change. *n* = 3 for control and Nrg1-ICD-expressing neurons. (b) The scatter plot shows the relative change in the mRNA expression of apoptotic genes in (a) grouped as pro- and antiapoptotic. *n* = 3 for control and Nrg1-ICD-expressing neurons.

Apoptotic-like pathways play an important role in the process of neuronal degeneration following cortical ischemia [41]. To investigate further the neuroprotective role of Nrg1-ICD, we measured the mRNA expression of a panel of candidate genes that control neuronal apoptosis by qPCR. These preliminary experiments suggest a role for Nrg1 intracellular signaling in regulating the expression of genes involved in apoptosis. While we failed to observe alterations to the expression of antiapoptotic genes such as Bcl-2 and Bcl2l1, we did discover a decrease in the expression of genes involved in proapoptotic signaling (caspases 2, 3, and 7 and Dapk1) (Figure 7). We speculate that the differential expression of pro- and antiapoptotic genes may render Nrg1-ICD-expressing neurons less susceptible to apoptotic stimuli, although future experiments will be required to ascertain the validity of this hypothesis.

### 3.8. Nrg1-ICD Is Neuroprotective upon Stroke *In Vivo*

Finally, we tested the relevance of Nrg1 intracellular signaling upon stroke *in vivo* by performing the stereotactic injection of AAVs expressing either Nrg1-ICD or GFP as a control in mouse brain cortices and then using a focal hemorrhagic stroke to provoke an infarct adjacent to the cortical region expressing Nrg1-ICD or GFP.

Briefly, we injected brain cortices with a small amount of collagenase (18 mU of collagenase type VII-S from *Clostridium histolyticum*) to weaken the extracellular matrix of the cerebral capillaries, thereby causing focal blood extravasation and hemorrhage. We allowed the animals to recover for 24 hours before being sacrificed.

**Figure 8:**
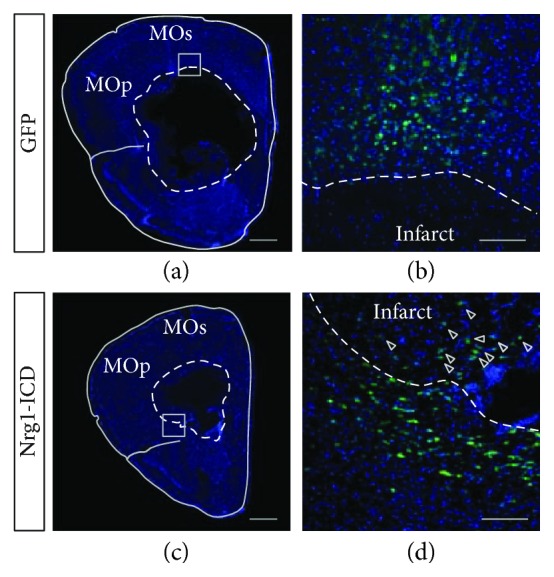
Nrg1-ICD is neuroprotective upon stroke *in vivo*. (a–d) Representative images of infarcted areas in the motor cortex of mice infected to express GFP as control (a, b) of Nrg1-ICD (c, d) at low (a, c) and high (b, d) magnification. GFP labeling (c, d) and counterstaining in DAPI (a, d). The dotted lines indicate the core of the infarcted regions. Arrowheads indicate surviving neurons expressing Nrg1-ICD inside the infarct (d). The boxed regions in (a) and (c) depict the areas magnified in (b) and (d), respectively. Scale bar in (a, c), 500 *μ*m. MOp: primary motor cortex; MOs: secondary motor cortex. Scale bar in (b, d), 100 *μ*m.

We analyzed the infarcted areas and scored the survival of neurons expressing Nrg1-ICD in comparison to controls in the core and the peri-infarct area (Figure 8 and Supplemental [Supplementary-material supplementary-material-1]). In the control cortices, we encountered neurons expressing GFP in the penumbra but we failed to observe GFP-positive neurons in the core of the infarct, as expected. Conversely, in cortices infected with Nrg1-ICD virus, we observed a limited but significant number of neurons expressing Nrg1-ICD in the core of the infarcted area in different sections (GFP, 0 out 13 positive sections analyzed in three brains; Nrg1-ICD, 24 out of 41 positive sections out of three animals; *p* < 0.01; Fisher's exact test). Even given the limited number of surviving neurons, these data provide proof-of-principle for a neuroprotective role for Nrg1 intracellular signaling following hemorrhagic cortical stroke *in vivo*.

## 4. Discussion

Nrg1 is a major regulator of cortical circuit development; however, we know relatively little regarding its role in mature neurons. In particular, the role of Nrg1 intracellular signaling remains largely unaddressed. Here, we investigated the role of this signaling pathway in neuroprotection and we now provide evidence that Nrg1 intracellular signaling can alleviate neuronal loss upon stroke in cortical neurons.

The expression of multiple Nrg1 isoforms and bidirectional signaling by Nrg1 combine to complicate the study of Nrg1 signaling. While we know little about the specific functional properties of each isoform, most of the isoforms expressed in the adult brain cortex (mainly types II and III) share the ICD that is released upon proteolytic processing to elicit Nrg1 intracellular signaling. Our data indicates the phylogenetic conservation of the regions that regulate the activation of NRG1 intracellular signaling and the nuclear localization of Nrg1-ICD, supporting the relevance of this signaling pathway. Also, our bioinformatic analyses suggest that NRG1 mRNA expression decreases during aging. Given previous studies that implicate Nrg1 in age-related diseases such as neuroinflammation, neurodegeneration, and stroke [14–16, 18–24], we assessed whether the decreased expression of Nrg1 might indicate a role of Nrg1 in neuroprotection. To examine the specific role of Nrg1 intracellular signaling, we took advantage of an experimental paradigm that we and others have successfully previously employed: we expressed the ICD to mimic the end product of Nrg1 proteolytic processing [5, 6]. To this end, we generated and validated new viral vectors to activate Nrg1 intracellular signaling more efficiently.

As Nrg1 expression decreased with aging, our first working hypothesis stated that Nrg1 intracellular signaling might be involved in senescence. To gain a preliminary insight into the role of Nrg1 in senescence, we tested whether Nrg1 intracellular signaling modulated the activation of p38 MAPK or DNA damage in an *in vitro* model of neuronal senescence. Various pathways associated with aging in neurons, such as inflammation and oxidative stress, activate p38 MAPK signaling [42]. Similarly, DNA damage in the brain leads to cellular senescence [42]. In our *in vitro* experimental paradigm, Nrg1 intracellular signaling did not regulate these neuronal senescence hallmarks. However, these observations do not exclude Nrg1 intracellular signaling from being involved in other aspects of aging in cortical neurons or under different experimental conditions and further studies will be required to investigate this hypothesis.

We next tested the hypothesis that Nrg1 intracellular signaling may be involved in stroke. Stroke is the second cause of death and disability worldwide, and as the risk of brain stroke increases with age, the societal burden of cortical infarct will increase significantly in the coming decades [41]. Interestingly, previous studies suggested a role for Nrg1 in stroke: Nrg1 expression increased upon stroke and the administration of the Nrg1-EGF domain alleviated experimental stroke [21–23]. To the best of our knowledge, the role of the Nrg1 intracellular signaling in stroke remains uninvestigated. Here, we established that OGD conditions trigger the activation of Nrg1 intracellular signaling, and as a result, Nrg1-ICD accumulates in the nucleus.

Moreover, our data indicate the neuroprotective function of Nrg1 intracellular signaling *in vitro* and *in vivo*. The expression of Nrg1-ICD delayed the onset of neural degeneration and improved neuronal survival *in vitro*. To address the role of Nrg1-ICD signaling *in vivo*, we employed collagenase injection as an experimental model of focal hemorrhagic stroke. Compared to other experimental setups, this model has the advantage of the controlled size and location of stroke by stereotactic injection. Thus, we provoked stroke in an area adjacent to the region previously infected to express Nrg1-ICD or GFP as control. Our data indicated that the activation of Nrg1 intracellular signaling improved neuronal survival *in vivo*. These findings support previous studies that indicate a crucial role for Nrg1 in stroke and unveil a novel function of Nrg1 intracellular signaling in neuroprotection in this pathology.

Mechanistically, the molecular machinery underlying Nrg1 intracellular signaling also remains largely unknown. In particular, the direct molecular interactors of Nrg1-ICD remain poorly understood. The Nrg1-ICD can interact with LIM domain kinase 1 (LIMK1) *in vitro* [43], while the overexpression of type I NRG1*β* leads to the enrichment and activation of LIMK1 in the synaptosomes, with this activation involved in synaptic transmission [44]. Consistently, studies have highlighted LIMK1 localization to the plasma membrane and neuronal synapses, and not the nucleus [45]. Therefore, LIMK1 may interact with the unprocessed membrane-bound form of Nrg1. It is unlikely that LIMK1 may be involved in the nuclear signaling of Nrg1-ICD.

The fact that the Nrg1-ICD sequence does not contain overt DNA binding or transcriptional motifs has complicated the understanding of Nrg1 nuclear signaling [6]. In spiral ganglion neurons, synaptic activity induces a transient interaction of Nrg1-ICD with the transcription factor Eos [46]. Therefore, hypoxic conditions may also induce the interaction of Nrg1-ICD with Eos or other unidentified transcription factors. Moreover, various mechanisms regulate gene expression, including chromatin remodeling or the regulation of mRNA stability by miRNA. Independently of the molecular interactors, our preliminary data suggest that Nrg1 intracellular signaling controls the apoptotic response, consistent with the observation that Nrg1 signaling promotes the survival in sensory ganglia [6]. However, the complex molecular biology of neurodegeneration in stroke remains poorly understood and we may envision different mechanisms. Many stroke risk factors promote the production of ROS [41]. For instance, glutamate excitotoxicity leads to accumulation of Ca^2+^, which in turn promotes the generation of ROS by inducing mitochondrial depolarization and the activation of ROS-generating enzymes [41]. Oxidative stress can promote inflammation, activate death signaling pathways, and inhibit synaptic activity.

As the Nrg1-ICD promotes neurotransmission and the formation of excitatory connections [5], we speculate that Nrg1-ICD might protect neurons from the synaptic dysfunction provoked by oxidative stress. We hope that future studies will determine the mechanisms underlying the neuroprotective role of Nrg1 intracellular signaling in this context.

In conclusion, our work establishes a novel role for intracellular Nrg1 signaling in neuroprotection upon stroke *in vitro* and *in vivo*. The finding that the highly conserved Nrg1-ICD has a neuroprotective role may foster the identification of new therapeutic targets to treat neurodegeneration following brain infarct.

## Data Availability

The data and reagents used to support the findings of this study are readily available from the corresponding author upon request.

## Supplementary Materials

Supplementary Materials Supplemental Figure S1: low levels of p38 MAPK activation and DNA damage at D14. (a) Representative images of neurons at D14 cultured under standard conditions and stained for phospho-p38 MAPK and DAPI. Immunostaining and imaging were performed under the same conditions as for D21 neurons in Figure 3. The bar on the right depicts the LUT for the signal intensity. Scale bar, 20 *μ*m. (b) Representative images of primary neurons as in (a), labeled with DAPI and phospho-H2AX staining. Immunostaining and imaging were performed under the same conditions as for D21 neurons in Figure 3. The bar on the right depicts the LUT for signal intensity. Scale bar, 20 *μ*m. (c) The graph illustrates the quantification of phospho-p38 MAPK labeling expressed in arbitrary units (a.u.). *n* = 4 fields. Average ± sem. (d) The graph illustrates the quantification of phospho-H2AX labeling expressed as a percentage of positive neurons. *n* = 4 fields. Average ± sem. Supplemental Figure S2: identification of the infarcted area. (a) Representative images of infarcted areas in the motor cortex from Figure 8 stained for DAPI and visualized in grey. Brains were infected to express GFP as control or Nrg1-ICD. Boxed areas are magnified in (c) with the corresponding numbers. The dotted line delimits the infarct area characterized by condensed pyknotic nuclei. Scale bar, 100 *μ*m. (b) The color code employed in the figure: red box for the infarcted area, green box for the noninfarcted area, blue for total DNA in the *mask* and graph, and magenta for condensed DNA in the mask and graph. (c) Magnified areas from (a). The mask shows the total area occupied by DNA in blue and the condensed DNA in magenta defined by an upper threshold of intensity. Scale bar, 25 *μ*m. The graph exhibits the quantification of the masks in the upper lane expressed as the total area occupied by the DNA (Tot, the fraction of the area of the image, in blue) and condensed DNA (Cond, the fraction over the total area of the DNA, magenta). In the infarcted area, the total area occupied by DNA decreases while the fraction of condensed DNA increases.
