# Supplementary figures and images for: Nrg1 Intracellular Signaling Is Neuroprotective upon Stroke
Source: Oxid Med Cell Longev. 2019 Sep 8;2019:3930186. doi: 10.1155/2019/3930186 (PMC6754950; doi:10.1155/2019/3930186)

Supplemental Figure S1

a

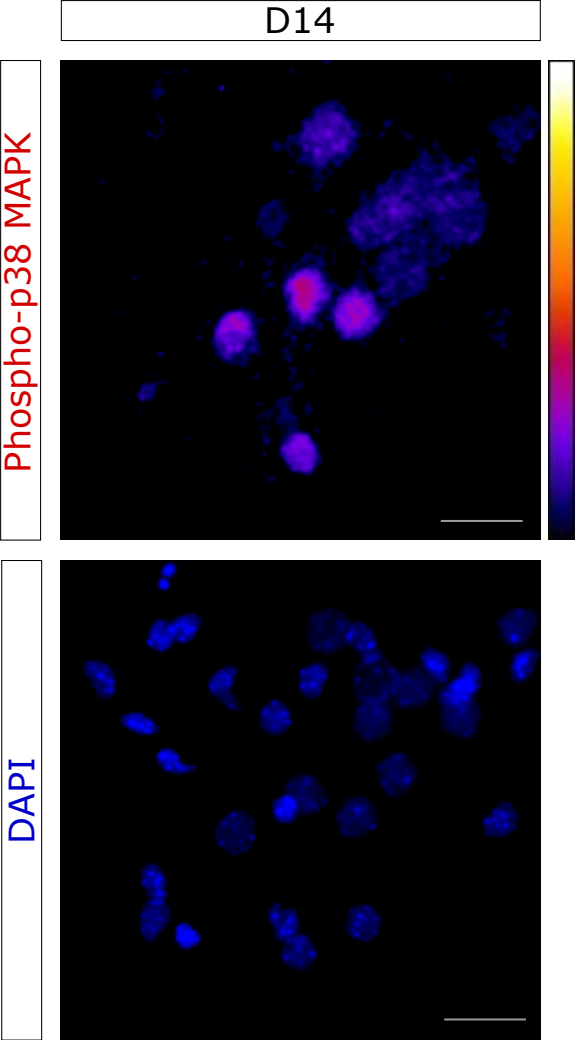

b

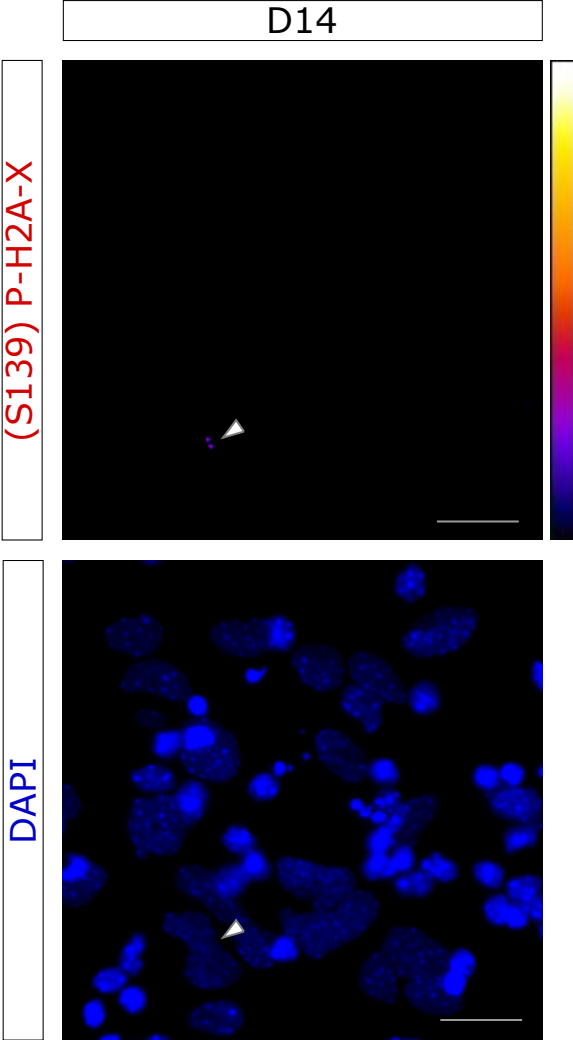

c

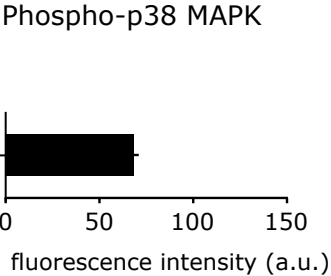

d

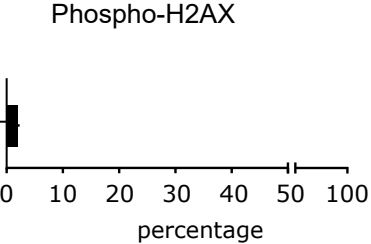

# Supplemental Figure S2

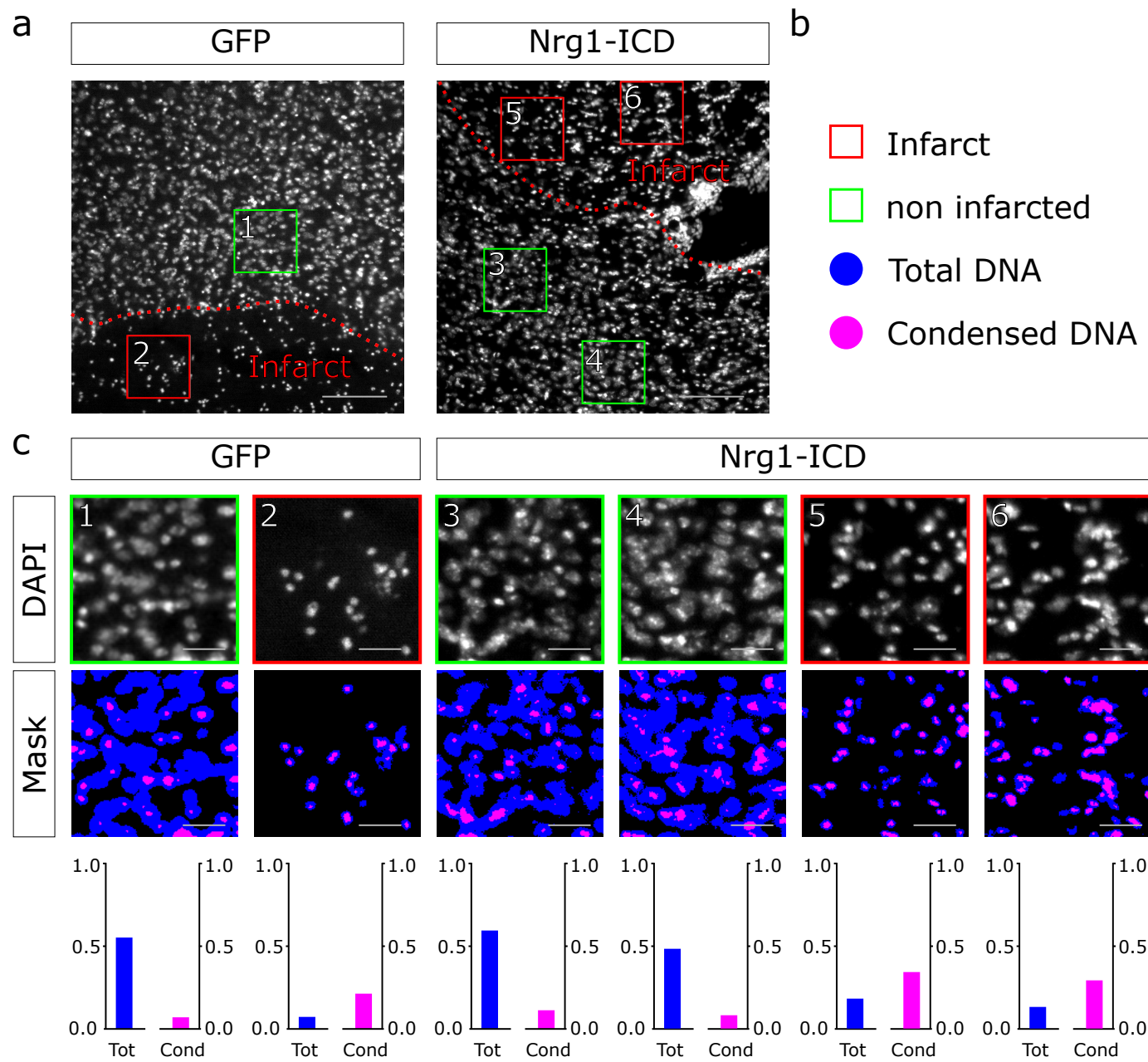

Supplement: Supplementary Materials — Supplemental Figure S1: low levels of p38 MAPK activation and DNA damage at D14. (a) Representative images of neurons at D14 cultured under standard conditions and stained for phospho-p38 MAPK and DAPI. Immunostaining and imaging were performed under the same conditions as for D21 neurons in Figure 3. The bar on the right depicts the LUT for the signal intensity. Scale bar, 20 μm. (b) Representative images of primary neurons as in (a), labeled with DAPI and phospho-H2AX staining. Immunostaining and imaging were performed under the same conditions as for D21 neurons in Figure 3. The bar on the right depicts the LUT for signal intensity. Scale bar, 20 μm. (c) The graph illustrates the quantification of phospho-p38 MAPK labeling expressed in arbitrary units (a.u.). n = 4 fields. Average ± sem. (d) The graph illustrates the quantification of phospho-H2AX labeling expressed as a percentage of positive neurons. n = 4 fields. Average ± sem. Supplemental Figure S2: identification of the infarcted area. (a) Representative images of infarcted areas in the motor cortex from Figure 8 stained for DAPI and visualized in grey. Brains were infected to express GFP as control or Nrg1-ICD. Boxed areas are magnified in (c) with the corresponding numbers. The dotted line delimits the infarct area characterized by condensed pyknotic nuclei. Scale bar, 100 μm. (b) The color code employed in the figure: red box for the infarcted area, green box for the noninfarcted area, blue for total DNA in the mask and graph, and magenta for condensed DNA in the mask and graph. (c) Magnified areas from (a). The mask shows the total area occupied by DNA in blue and the condensed DNA in magenta defined by an upper threshold of intensity. Scale bar, 25 μm. The graph exhibits the quantification of the masks in the upper lane expressed as the total area occupied by the DNA (Tot, the fraction of the area of the image, in blue) and condensed DNA (Cond, the fraction over the total area of the DNA, magenta). In [file 3930186.f1.pdf]
